# A False Step in a Ballerina: A Rare Case of Osteonecrosis of Subhallucal Sesamoid Bone

**DOI:** 10.7759/cureus.72766

**Published:** 2024-10-31

**Authors:** Pedro Maganinho, Filipe Sá Malheiro, Joana Cardoso, Carlos Sampaio Macedo, Bruno S Pereira

**Affiliations:** 1 Radiology, Centro Hospitalar Universitário do Porto, Porto, PRT; 2 Orthopedics and Traumatology, Hospital Lusíadas Braga, Braga, PRT; 3 Orthopaedics and Traumatology, Clínica Espregueira, Porto, PRT; 4 ICVS Life and Health Sciences Research Institute, Universidade do Minho, Braga, PRT; 5 Radiology, Hospital Lusíadas Braga, Braga, PRT; 6 Radiology, Hospital Lusíadas Porto, Porto, PRT; 7 Radiology, Clínica Espregueira, Porto, PRT; 8 Orthopaedics and Traumatology, Unidade Local de Saúde de Barcelos/Esposende, Braga, PRT

**Keywords:** ct scan, mri, osteonecrosis, sesamoid, subhallucal

## Abstract

Subhallucal interphalangeal osteonecrosis is an uncommon cause of forefoot pain, and a rarely reported clinical entity, being often overlooked. Imaging, particularly computed tomography (CT) scan and magnetic resonance imaging (MRI) have an essential role in early and differential diagnosis and guiding for appropriate therapy. The first approach should be conservative, and surgical treatment should be considered when it proves ineffective. In this case report, we describe a rare case of hallucal interphalangeal osteonecrosis manifesting a long-term hallux discomfort, successfully managed through a conservative approach, and we discuss the role of imaging techniques in its diagnosis.

## Introduction

Forefoot pain is a frequent complaint encountered in daily practice. While sesamoid bone pathologies are a potential cause, they are rarely diagnosed; however, they account for approximately 9% of foot and ankle injuries[1,2]. Sesamoid bones are small and may appear functionally negligible, but they play a crucial role in supporting the weight-bearing function of the foot and the hallux. Their pathologies may result in severe pain and disability [3]. Osteonecrosis of the sesamoid bones is a rare entity, and its diagnosis may be challenging [4]. Avascular osteonecrosis of the metatarsal sesamoids is a classic, first described by Renander et al., although a rare condition of the hallux interphalangeal sesamoid bones [5-7]. By altering the biomechanics of the movements at great toe, the interphalangeal sesamoid becomes susceptible to pathologies like trauma, infection, degeneration, inflammation, and osteonecrosis. Physical examination and imaging methods are useful in the diagnosis. Insufficient recognition of this condition results in misdiagnosis and delayed treatment, ultimately contributing to significant morbidity [5].

Our aim was to report a rare case of osteonecrosis of the subhallucal sesamoid bone, discuss the role of image techniques in its diagnosis, share our approach, and review the literature regarding this issue.

## Case presentation

We report a case of a 22-year-old female patient, a ballerina by occupation with no past medical history and chronic medication, who presented to our outpatient clinic with a history of mechanical right forefoot pain. The pain started one year ago and was located on the plantar aspect of the great toe, primarily at the interphalangeal joint, mostly when weight was given on the great toe, mostly at the tiptoe dancing movements that incapacitated the ballerina from performing. The pain got worse and, after a few months and was present even when walking. The patient had no history of acute recent trauma. 

**Figure 1:**
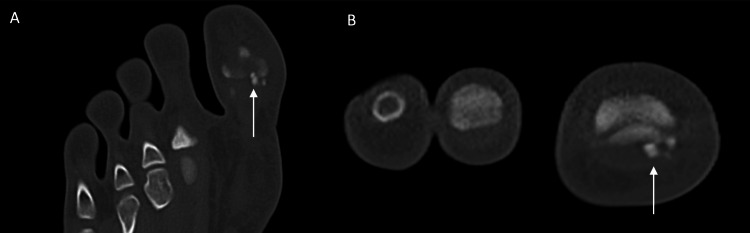
CT coronal (A) and axial (B) images showing sclerosis of subhallucal sesamoid bone (white arrows)

On clinical examination, this site was palpated with severe tenderness, with no inflammatory signs like redness or swelling. The passive and active movements were normal but painful. No sensory or motor deficit was found. Laboratory analysis and anteroposterior and lateral radiographs of the foot were normal. A CT examination revealed increased sclerosis in the medial (tibial) subhallucal sesamoid bone at the interphalangeal joint, consistent with osteonecrosis (Figure 1).

**Figure 2:**
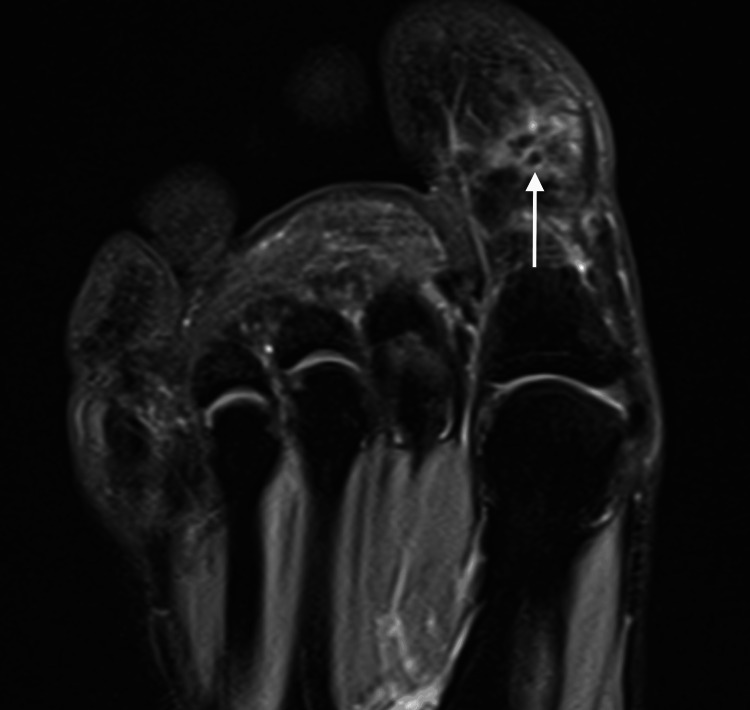
Magnetic resonance imaging. T2-weighted coronal image reveal small subhallucal sesamoid bone (white arrow) with hypointense bone marrow and edema of the adjacent fat planes

In MRI, the subhallucal sesamoid bone showed decreased size and signal and edema of the adjacent fat planes, consistent with osteonecrosis (Figure 2). 

After the diagnosis of osteonecrosis of the sesamoid bone, under ultrasound guidance, the patient received a local injection of 1 ml of 1% lidocaine mixed with 10 mg of triamcinolone acetonide between the sesamoid bone and flexor hallux longus tendon. The pain ceased immediately. Currently, with approximately 3 months of follow-up, the patient remains asymptomatic.

## Discussion

The subhallucal interphalangeal sesamoid is an ossicle, with a portion of it being embedded within the flexor hallucis tendon at the interphalangeal joint of the great toe [4]. The reported incidence of subhallucal interphalangeal sesamoid bones is 2-13%, and in most cases, they remain asymptomatic [8-10]. Subhallucal interphalangeal sesamoid pathologies are rare conditions known to cause significant great toe pain, being often overlooked and misdiagnosed for unrelated conditions. These painful disorders may present with an acute post-traumatic or insidious onset and can have several etiologies like trauma, overuse, arthritis, infection, or ischemia [11,12]. The flexor hallucis longus tendon is the most affected due to its anatomy [11].

Avascular osteonecrosis of the subhallucal sesamoid bones is a very uncommon entity, and lack of knowledge can pose diagnostic challenges and lead to misdiagnosis, delayed treatment, and significant morbidity [4]. To the best of our knowledge, there are only a few cases of this pathology described in the literature [11]. While there are cases described in the literature, the majority involve other sesamoid bones of the foot, with the hallucal sesamoid being the most common [13-15]. Avascular osteonecrosis of the subhallucal sesamoid must be carefully differentiated from other etiologies that cause pain in this region, particularly sesamoiditis and fractures, with magnetic resonance imaging being the most suitable modality for distinction. In cases of avascular necrosis, the bone marrow exhibits hypointense signals on T1 and T2-weighted sequences. In contrast, both sesamoiditis and fractures demonstrate hyperintense signals on T2-weighted sequences, with the presence of a fracture line on T1-weighted sequences or more frequently on CT, serving as the distinguishing feature for fractures.

This case underscores the importance of a comprehensive clinical history, a thorough physical examination, and advanced imaging modalities, such as CT and MRI, to an accurate and definitive diagnosis. The findings of heterogeneous and abnormal shape bone structure, fragmentation and increased sclerosis on CT, and decreased size and signal in the subhallucal sesamoid bone on MRI, may be detected at the initiation of the necrotic process, allowing the diagnosis which are consistent with previous reports [13-15].

The most effective diagnostic tool for early detection is MRI, which allows for monitoring of the osteonecrotic process [15,16]. Osteonecrosis at early stages shows features of marrow edema, and at late stages, sclerosis predominates. In the early stages, the sesamoids appear hyperintense on STIR images and mildly hypointense on T1-weighted images. However, in the later stages, they are hypointense across all sequences due to sclerosis. Due to the fact that both osteonecrosis and early-stage sesamoiditis exhibit similar imaging characteristics of bone marrow edema, MRI lacks the reliability to effectively differentiate between these two conditions. CT can be a valuable tool in these cases since it demonstrates increased sclerosis in osteonecrosis and normal density in sesamoiditis [10]. Plain radiographs are usually normal in the acute phase but progressively can demonstrate occasionally heterogeneous striated sclerosis, flattening of the sesamoid bone, and demineralization [7,13]. Nevertheless, the small size and oval, rough, convex morphology of sesamoid bones, along with their composition of bone, cartilage, and fibrous tissue, frequently lead to their contours being obscured on radiographs by the radiopacity of larger adjacent bones [8,17]. Consequently, in instances of foot pain with an unclear origin, the subhallucal interphalangeal sesamoid bones must be regarded as a potential differential diagnosis, especially when radiographs yield normal or inconclusive results. In such cases, CT and MRI are essential diagnostic modalities.

We emphasize the importance of considering sesamoid bone pathologies, albeit rare, in the differential diagnosis of forefoot pain, particularly in individuals with predisposing factors such as high-impact activities in the forefoot, like ballet [14]. Clinicians should maintain a high index of suspicion for osteonecrosis in patients presenting with chronic great toe pain, especially in the absence of recent trauma. Moreover, increased awareness of this clinical entity among healthcare providers is crucial to prevent misdiagnosis, delayed treatment, and consequent morbidity. Although rare, the possibility of a pathological fracture should also be considered.

The successful conservative treatment approach employed in this case, involving a local injection under ultrasound guidance followed by oral nonsteroidal anti-inflammatory drugs and rest, highlights the potential efficacy of non-surgical management strategies in swiftly improving symptoms and promoting recovery. Shin HY et al. also demonstrated the efficacy of this treatment [11]. In addition to hydrotherapy, cryotherapy, and ultrasound, which are conservative treatments noted in cases of osteonecrosis of the hallucal sesamoids, the continuous use of orthotics or a cast may be considered for patients with severe symptoms [7,13,16]. Surgical treatment is recommended when conservative treatments are unsuccessful to reduce complication risk [17-19]. Further research is needed to compare the treatment strategies to elucidate the optimal treatment algorithm for subhallucal interphalangeal osteonecrosis, considering factors such as disease severity, patient preferences, and functional outcomes [11]. We also need a longer follow-up to understand the long-term efficacy and potential recurrence of symptoms of the ultrasound-guided injection.

In conclusion, this case report provides valuable clinical insights into the diagnosis and management of subhallucal interphalangeal osteonecrosis. It highlights that this is a rare and disabling pathology in which history, physical examination, CT, and MRI can be essential to prompt and refined diagnosis and that can rapidly respond to corticosteroid local injection. In cases where conservative treatments are ineffective, surgical options may be pursued.

## Conclusions

This case underscores the importance of interdisciplinary collaboration (orthopedics and radiology), advanced imaging modalities, and individualized treatment approaches. Clinics should be aware of this rare pathology to promptly perform appropriate diagnostic tests and treat accordingly in order to swiftly improve the quality of life and outcomes of patients and the rapid return to physical/work activities. Future research should include prospective studies to elucidate the natural history of subhallucal interphalangeal osteonecrosis, comparative trials evaluating the efficacy of conservative versus surgical management strategies, and investigations into potential risk factors and prognostic indicators for disease progression.

## References

[1] Bartosiak K, McCormick JJ (2019). Avascular necrosis of the sesamoids. Foot Ankle Clin.

[2] Boike A, Schnirring-Judge M, McMillin S (2011). Sesamoid disorders of the first metatarsophalangeal joint. Clin Podiatr Med Surg.

[3] Richardson EG (1999). Hallucal sesamoid pain: causes and surgical treatment. J Am Acad Orthop Surg.

[4] Kumar S, Kadavigere R, Puppala R, Ayachit A, Singh R (2015). Subhallucal interphalangeal sesamoiditis: a rare cause of chronic great toe pain. J Clin Diagn Res.

[5] Renander A (1924). Two cases of typical osteochondropathy of the medial sesamoid bone of the first metatarsal. Acta radiol.

[6] Huber-Levernieux C, Denis A (2003). Aseptic osteonecrosis of the metatarsal sesamoids or Renander's disease: current aspects about 2 cases. Rheumatism Review.

[7] Toussirot E, Jeunet L, Michel F, Kantelip B, Wendling D (2003). Avascular necrosis of the hallucal sesamoids update with reference to two case reports. Joint Bone Spine.

[8] Roukis TS, Hurless JS (1996). The hallucal interphalangeal sesamoid. J Foot Ankle Surg.

[9] Davies MB, Abdlslam K, Gibson RJ (2003). Interphalangeal sesamoid bones of the great toe: an anatomic variant demanding careful scrutiny of radiographs. Clin Anat.

[10] Nwawka OK, Hayashi D, Diaz LE (2013). Sesamoids and accessory ossicles of the foot: anatomical variability and related pathology. Insights Imaging.

[11] Shin HY, Park SY, Kim HY, Jung YS, An S, Kang DH (2013). Symptomatic hallucal interphalangeal sesamoid bones successfully treated with ultrasound-guided injection: a case report. Korean J Pain.

[12] Mellado JM, Ramos A, Salvadó E, Camins A, Danús M, Saurí A (2003). Accessory ossicles and sesamoid bones of the ankle and foot: imaging findings, clinical significance and differential diagnosis. Eur Radiol.

[13] Fleischli J, Cheleuitte E (1995). Avascular necrosis of the hallucial sesamoids. J Foot Ankle Surg.

[14] Pinto R, Freitas D, Massada M, Gonçalves I, Muras J (2010). Hallux sesamoid osteonecrosis associated to ballet. Rev Port Ortop Traum.

[15] Williams G, Kenyon P, Fischer B, Platt S (2009). An atypical presentation of hallucial sesamoid avascular necrosis: a case report. J Foot Ankle Surg.

[16] Aşkın A, Güvendi E, Tosun A, Tosun Ö (2017). Osteonecrosis of the sesamoid bones: two case reports. J Clin Anal Med.

[17] Del Rossi G (2003). Great toe pain in a competitive tennis athlete. J Sports Sci Med.

[18] Coughlin MJ (2007). Sesamoids and accessory bones of the foot. Surgery of the Foot and Ankle.

[19] Valinsky MS, Hettinger DF, Mortvedt JK (1989). Hallux interphalangeal joint sesamoidectomy using minimal incision technique. J Am Podiatr Med Assoc.

